# Supplementary material for: Time‑resolved multi-omic analysis of paclitaxel exposure in human iPSC‑derived sensory neurons unveils mechanisms of chemotherapy‑induced peripheral neuropathy
Source: Cell Death Dis. 2026 Feb 10;17(1):211. doi: 10.1038/s41419-026-08445-2 (PMC12921266; doi:10.1038/s41419-026-08445-2)
Supplement: Supplementary file 15 — Dataset 1. [file 41419_2026_8445_MOESM15_ESM.zip › CDD_Open_Data/04_RNA_Seq/Timeline_panel_plots_etc_2023-01-23_1609.zip!/Panel_Plots_etc/09_tmod_panel_plot_msigdb_kegg_pval_filter_0.01_pval0.05_AUC0.7_L2FC0.5.pdf]

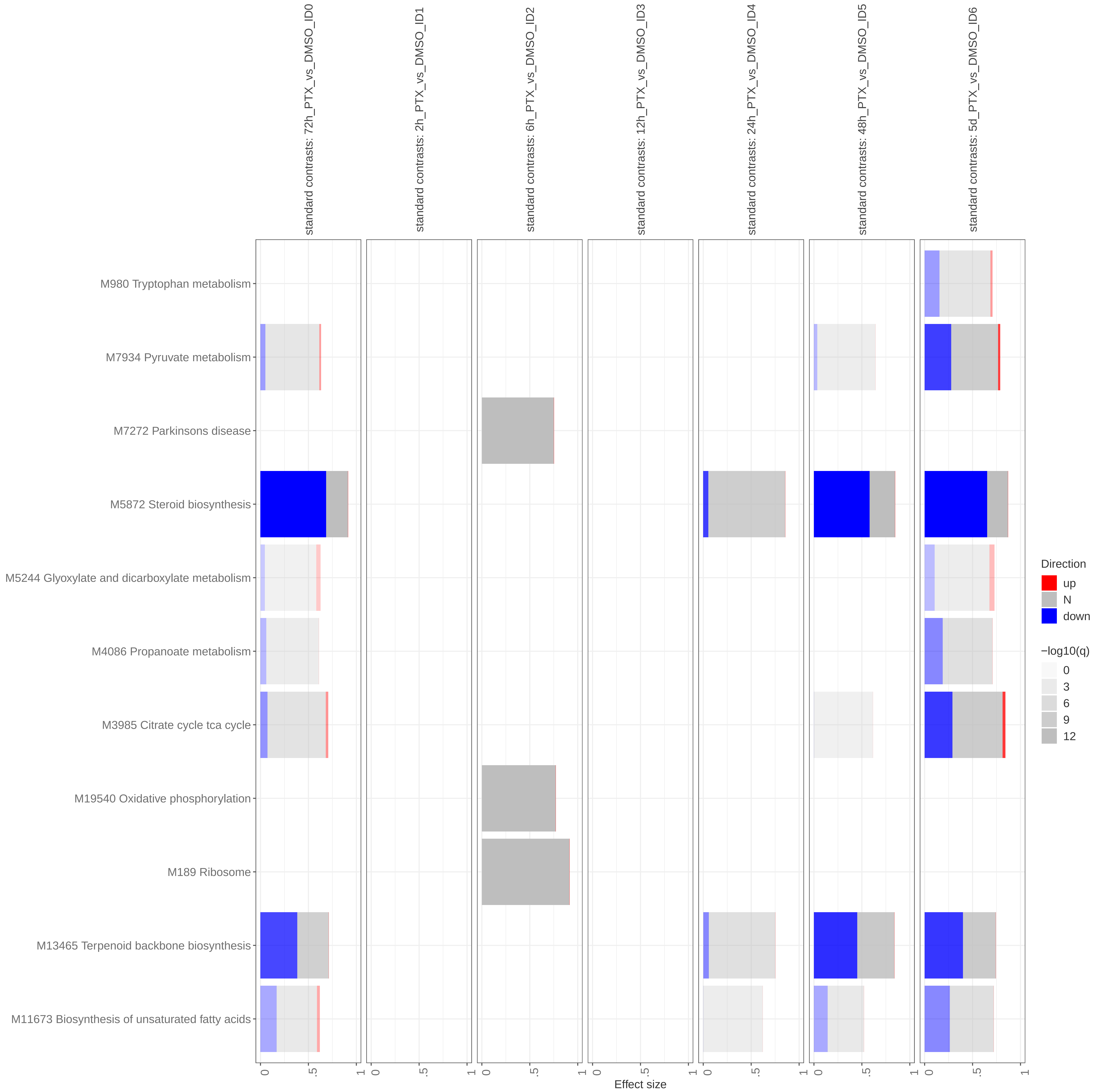

standard contrasts: 72h_PTX_vs_DMSO_ID0
standard contrasts: 2h_PTX_vs_DMSO_ID1
standard contrasts: 6h_PTX_vs_DMSO_ID2
standard contrasts: 12h_PTX_vs_DMSO_ID3
standard contrasts: 24h_PTX_vs_DMSO_ID4
standard contrasts: 48h_PTX_vs_DMSO_ID5
standard contrasts: 5d_PTX_vs_DMSO_ID6
M980 Tryptophan metabolism
M7934 Pyruvate metabolism
M7272 Parkinsons disease
M5872 Steroid biosynthesis
M5244 Glyoxylate and dicarboxylate metabolism
M4086 Propanoate metabolism
M3985 Citrate cycle tca cycle
M19540 Oxidative phosphorylation
M189 Ribosome
M13465 Terpenoid backbone biosynthesis
M11673 Biosynthesis of unsaturated fatty acids
0
.5
1
Effect size
Direction
up
N
down
−log10(q)
0
3
6
9
12
